# The reconstruction of fingertip injury by mini hallux toenail flap pedicled with the hallux transverse artery and toe pulp vein transplantation technique based on the equivalent design theory

**DOI:** 10.1186/s12893-023-02097-1

**Published:** 2023-08-11

**Authors:** Xiao Fan, Yimin Zhou, Jian Zhou, Shiyou Dai, Jinhai Liu, Kecheng Lao

**Affiliations:** 1 Qingdao Hospital, University of Health and Rehabilitation Science (Qingdao Municipal Hospital), Qingdao, 266011 Shandong China; 2 Changchun University of Traditional Chinese Medicine, Changchun, Jilin, 130117 China

**Keywords:** Fingertip injury, Toenail flap, Free transplantation, Plastic surgery, Equivalent design theory

## Abstract

**Introduction:**

How to reconstruct the damaged fingertip is a clinical problem. Our team propose the theory of equivalent design and use the mini toenail flap pedicled with the hallux transverse artery and toe pulp vein transplantation technique to reconstruct Allen’s type II fingertip injury. Thus, we perform the retrospective study to evaluate the effects of this technique on fingertip injury.

**Materials and methods:**

A retrospective analysis was performed on 56 patients admitted to our hospital from January 2015 to January 2020 who used equivalently designed miniature hallux toenail flaps for the plastic repair of fingertip damage. We recorded the size of the miniature hallux toenail flap, operation time, intraoperative blood loss, and complications and calculated the survival rate of the transplanted miniature hallux toenail flap. During routine follow-up after surgery, we recorded nail growth time and observed finger appearance. At the last time of follow-up, we recorded Semmes-Weinstein evaluating tactile sensation and Two-point discrimination testing (TPD). The efficacy was evaluated by Zook score evaluation.

**Results:**

The size of the mini hallux toenail flap was 0.71 cm × 1.22 cm to 0.88 cm × 1.71 cm. The operation time was (3.54 ± 0.58) hours, the intraoperative blood loss was (20.66 ± 4.87) ml, and the survival rate of mini hallux toenail flaps was 100%. The postoperative follow-up time was (30.82 ± 11.21) months, and the total nail growth time was (9.68 ± 2.11) months. The average tactile sensation evaluated by the Semmes-Weinstein test was (0.32 ± 0.14) g, and the average TPD was (7.33 ± 1.02) mm. According to Zook score, the curative effect of fifty-six cases were all excellent or good with 100% excellent and good rate, and all patients had beautiful appearances and good function of damaged fingertips.

**Conclusions:**

Based on the equivalent design theory, the mini hallux toenail flap pedicled with the hallux transverse artery and toe pulp vein transplantation technique is an effective method to reconstruct Allen’s type II fingertip injury with a beautiful appearance and good function.

**Type of study/level of evidence:**

Therapeutic IV.

## Introduction

Fingertip injury is a common hand injury caused by machines in clinics. The soft tissue loss caused by fingertip injury not only affects the appearance of fingers but also seriously influences the function of fingers, which seriously impacts the life, work, and psychology of patients. Currently, the clinical treatments for fingertip injury mainly include conservative treatments and surgical treatments. For conservative treatments, irrigation to the wound with saline and dressing are the main modalities with some advantages as simple option, less pain, and low cost [1]. But with no effect on repairing missing tissue and nail bed, conservative treatments have a high probability of causing shortened digits, nail loss or shortening, leading to an ugly appearance of the damaged finger. Surgical treatments include random abdominal flap, local pedicle flap, advancement flap, and other surgeries to repair the fingertip injury with the advantage of simple operation. Nevertheless, these methods may cause swollen fingertips with no nails or apparent differences in nail color and poor fingertip function in patients [2–4]. Therefore, how to reconstruct the damaged fingertip, including skin and nail bed, and restore fingertip function is a clinical problem.

Studies have shown that [2–5] toenail flap transplantation is an effective method to repair fingertip injury. Currently, most toenail flaps in clinics use the toe base artery, the toe dorsal artery, or the first toe dorsal artery as the arterial blood supply system and the toe dorsal vein as the venous return system [5–8]. In order to increase the venous return of these transplanted toenail flaps, the surgeons must carry some redundant skin pedicle when removing the toenail flaps. On the one hand, these toenail flaps inevitably increase donor site damage. On the other hand, they lead to a relatively swollen finger after surgery, resulting in an ugly repaired finger. Therefore, it is a clinical problem for hand surgeons to skillfully design the miniature toenail flap for transplantation which can reduce the donor site injury and repair the fingertip injury aesthetically.

The theory of “equivalent design” is a new theory proposed by our team based on the design concept of “what is missing, what is needed, how much is missing, and how much is needed”. Based on the theory, we take the hallux toenail pulp and nail bed as donors, and by evaluating the defect of finger pulp and nail bed, we acquire mini hallux toenail flap pedicled with the hallux transverse artery and toe pulp vein from the donors to reconstruct damaged fingertip, which is different to previous operations. Its theoretical core is to accurately design the size and shape of the donor area with the damaged area as the template, without redundant tissue to accurately reconstruct the damaged fingertip, which can restore the normal appearance and function of the damaged fingertip.

## Materials and methods

**Fig. 1:**
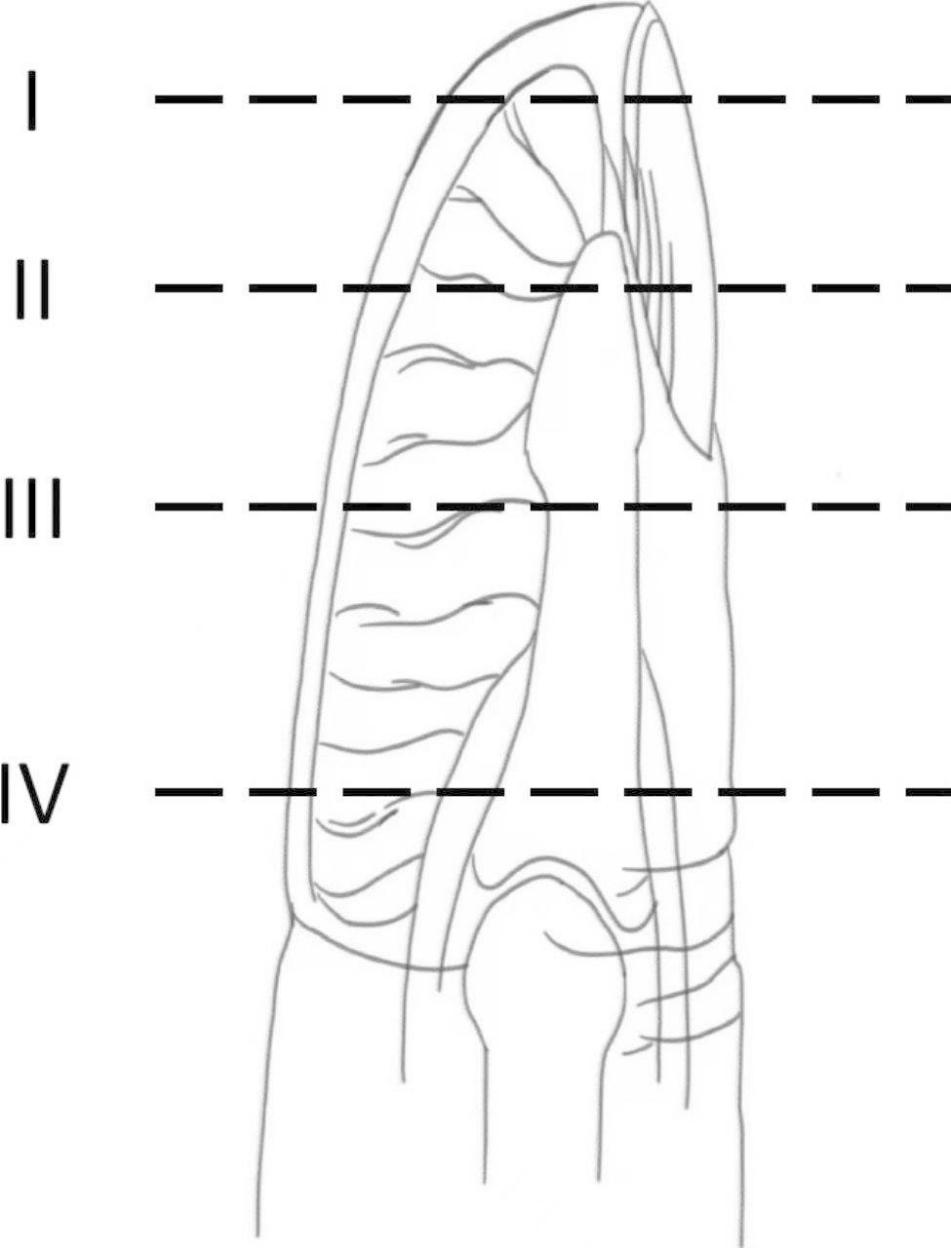
Allen’s classification of fingertip injury. Type **I**: Only finger pulp is injured. Type **II**: The finger pulp with less than 1/2 of the nail bed and a small amount of the distal phalanx are injured. Type **III**: Entire nail bed and the partial distal phalanx are injured. Type **IV**: Proximal nail fold and distal phalanx are injured

General information: The work is on a retrospective study of fifty-six patients with fingertip injuries subjected to miniature hallux toenail flap pedicled with the hallux transverse artery and toe pulp vein transplantation based on the “equivalent design” theory from January 2015 to January 2020. According to Allen’s classification of fingertip injury, all fingertip injuries included in the study were Allen’s type II (Fig. 1). Among the fifty-six patients with fingertip injury, thirty-six patients were male, and twenty were female, aged (40.15 ± 5.44) years. Among the fifty-six patients, fifty-six fingertips were injured, including twenty thumbs, seventeen indicator fingers, ten middle fingers, and nine ring fingers. Thirty-six cases were caused by door crush injuries, and twenty cases were caused by machine crush injuries. Soft tissues defected from 0.60 cm × 1.00 cm to 0.8 cm × 1.5 cm, and nail bed defected from 0.40 cm × 0.70 cm to 0.60 cm × 0.80 cm. The time from injury to operation was (4.78 ± 1.35) hours, and all patients received emergency operations. (Table 1)

**Table 1:** General information of patients included in the study

Number	Gender (M/F)	Age(years)	Thumb/Index finger/Middle finger/Ring finger/Little finger	Soft tissue defect area (cm^2^)	Nail bed defect area (cm^2^)	Time from injury to operation (hours)
56	36/20	40.15 ± 5.44	20/17/10/9	0.60 to 1.2	0.28 to 0.48	4.78 ± 1.35

The study has been approved by Qingdao Municipal Hospital Ethics Committee, and all patients participating in the study have signed the informed consent form.

Inclusion criteria: (i) Allen’s type II fingertip injury as the fingertip beyond the semilunar line is damaged with partial defects of the nail bed and pulp, complete or slightly missing distal phalanx, and complete nail root. (ii) Less than 60 years old with strong demand for reconstruction of the fingertip. (iii) Without diseases affecting the quality of vascular anastomoses, such as diabetes, rheumatism, etc. (iv) No gross contamination on the wound surface. (v) Without mental illness, follow the doctor’s advice and follow-up.

Exclusion criteria: (i) Nail root injury. (ii) Serious phalangeal fractures. (iii) The end of the finger is completely missing. (iv) Those who cannot quit smoking and drinking and cooperate with the treatment. (v) Patients with degloving injury of the distal finger segment.

### Surgical technique

**Fig. 2:**
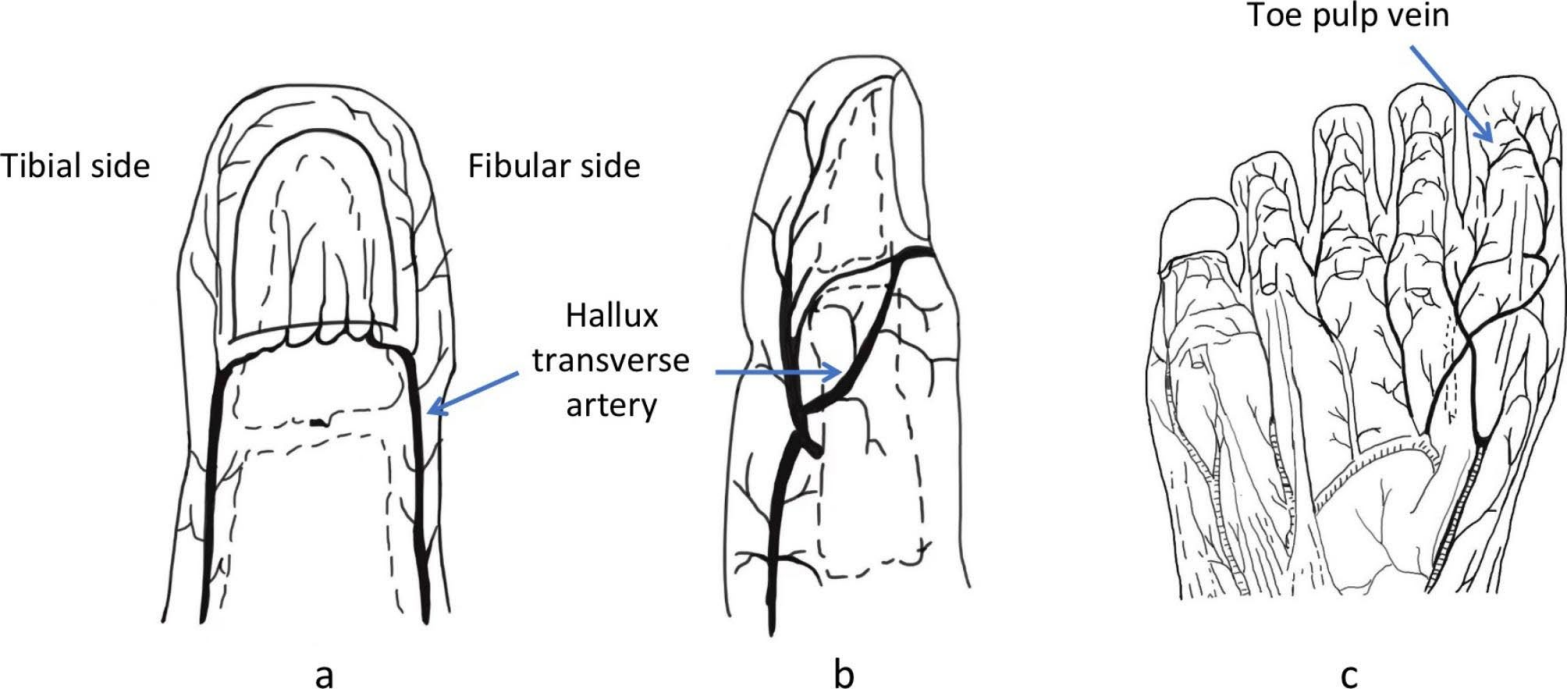
Anatomy of hallux transverse artery and toe pulp vein. **(a)** Anatomy of hallux transverse artery on AP view. **(b)** Anatomy of hallux transverse artery on the lateral view. **(c)** Anatomy of toe pulp vein on AP of the toe pulp

The patient was placed in the supine position, the surgical area was disinfected, and towels were laid. After successful finger or toe root anesthesia, two surgeons performed hand debridement to separate the finger ventral vein and digital artery. At the same time, another two surgeons performed the “equivalent design” to acquire the mini hallux toenail flap pedicled with the hallux transverse artery and toe pulp vein as follows: Under the microscope, the surgeons split the nail bed 2 mm outside the nail groove on the fibular side of the hallux in the donor area until to the trochanter bone surface of the toe and then exposed and dissociated the hallux transverse artery for the length of 5 to 10 mm, and 1 or 2 toe pulp vein with 5 to 10 mm length at the corresponding position according to the anatomy which was taken according to the venous position marked by the finger recipient site (Fig. 2). Cutting the toe ventral skin along the design pattern, the surgeons cut the nail bed closely attached to the periosteum of the toe bone by a combination of anterograde and retrograde methods to obtain a completely free mini hallux toenail flap pedicled with the hallux transverse artery and toe pulp vein. The shape and placement direction of the mini hallux toenail flap shall be designed according to the shape of the damaged fingertip (for example, if the defect is horizontal, the mini hallux toenail flap shall be placed horizontally; if the defect is oblique, the mini hallux toenail flap shall be placed obliquely) and cover the recipient site with the skin of the mini hallux toenail flap covering the skin defect and the nail bed of mini hallux toenail flap covering the nail bed defect separately. The nail bed stump of the mini hallux toenail flap should be repaired and sutured with the nail bed of the recipient site. Then the hallux transverse artery was anastomosed with the digital artery, and the toe pulp vein was anastomosed with the digital ventral vein. Lastly, the donor area was sutured directly.

### Postoperative management

Postoperative management included the limiting movement of affected limbs, raising the affected limb, conventional micro-surgical treatments, routine dressing changes of the wound, and erythromycin ointment applied on the exposed nail bed to keep it moist.

### Follow-up and outcomes

The time of follow-up was (30.82 ± 11.21) months. Outcomes include the size of the mini hallux toenail flap, operation time, blood loss during operation, complications, the survival rate of mini hallux toenail flap, fingertip appearance and nail growth time, Zook score [9] evaluating nail repair, Semmes-Weinstein evaluating tactile sensation and Two-point discrimination testing (TPD).

## Results

The size of the mini hallux toenail flap was from 0.71 cm × 1.22 cm to 0.88 cm × 1.71 cm. The operation time was (3.54 ± 0.58) hours, and blood loss during operations was (20.66 ± 4.87) ml. The vascular crisis occurred in three mini hallux toenail flaps, including one venous and two arterial crises, which all survived after surgical treatment. All fifty-six cases of min hallux toenail flap survived with a 100% survival rate. There were no complications such as infection, pain, and paresthesia in the donor site of fifty-six patients, and there was no obvious abnormality in walking function. At the last follow-up, all of the damaged fingertips of fifty-six patients had beautiful appearances and recovered to normal fingertip and nail contour, basically without nail deformity. The average nail growth time of fifty-six patients was (9.68 ± 2.11) months. The average tactile sensation of fifty-six patients evaluated by the Semmes-Weinstein test was (0.32 ± 0.14) g, and the average TPD of fifty-six patients was (7.33 ± 1.02) mm. According to Zook score evaluating nail repair, the curative effect of fifty-six cases were all excellent or good, with a 100% excellent and good rate. Besides, all donor sites were sutured directly with a beautiful appearance, and there was no impact on walking for all patients.

**Table 2:** Summary table of results

Operation time(hours)	3.54 ± 0.58
Intraoperative blood loss(ml)	20.66 ± 4.87
Survival rate of mini hallux toenail flaps(%)	100
Postoperative follow-up time(months)	30.82 ± 11.21
Total nail growth time(months)	9.68 ± 2.11
Average tactile sensation, Semmes-Weinstein test(g)	0.32 ± 0.14
Average TPD, Semmes-Weinstein test(mm)	7.33 ± 1.02
Excellent-good rate of curative effect, Zook score(%)	100

### Case presentations

#### Patient I

**Fig. 3:**
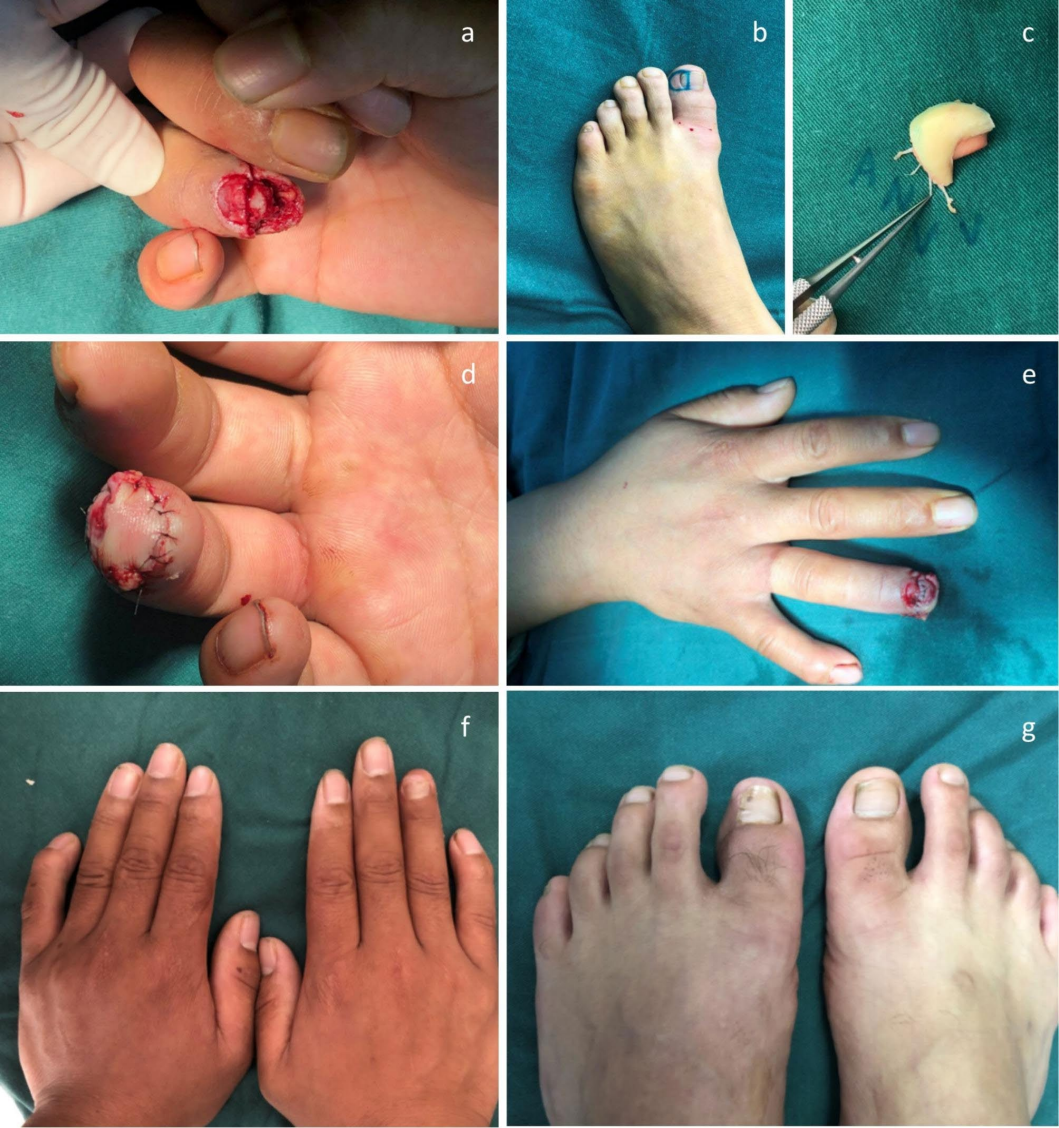
Mini hallux toenail flap pedicled with the hallux transverse artery and toe pulp vein for fingertip injury of the right ring finger. **(a)** Fingertip injury of the right ring finger (Allen’s type II). **(b)** Equivalent design of donor area. **(c)** Free mini hallux toenail flap pedicled with the hallux transverse artery and toe pulp vein for transplantation. **(d-e)** Mini hallux toenail flap pedicled with the hallux transverse artery and toe pulp vein was transplanted to cover the defects of skin and nail bed during the operation. **(f)** The appearance of the injured right ring finger 25 months after the operation. **(g)** The appearance of the donor area of the left hallux 24 months after the operation

A twenty-one years old male worker with his right ring fingertip damaged by a machine was sent to the emergency room within 3 h after injury. Emergency debridement was performed, finding that soft tissue defect and nail defect of the right ring fingertip was about 0.6 cm × 1.0 cm and 0.6 cm × 0.8 cm, respectively, with intact phalanx (Allen’s type II). Then the mini hallux toenail flap pedicled with the hallux transverse artery and toe pulp vein based on the “equivalent design” theory was used to reconstruct the damaged fingertip. The graft survived, and the damaged fingertip and donor site were beautiful, with good function 25 months after surgery. (Fig. 3)

### Patient II

**Fig. 4:**
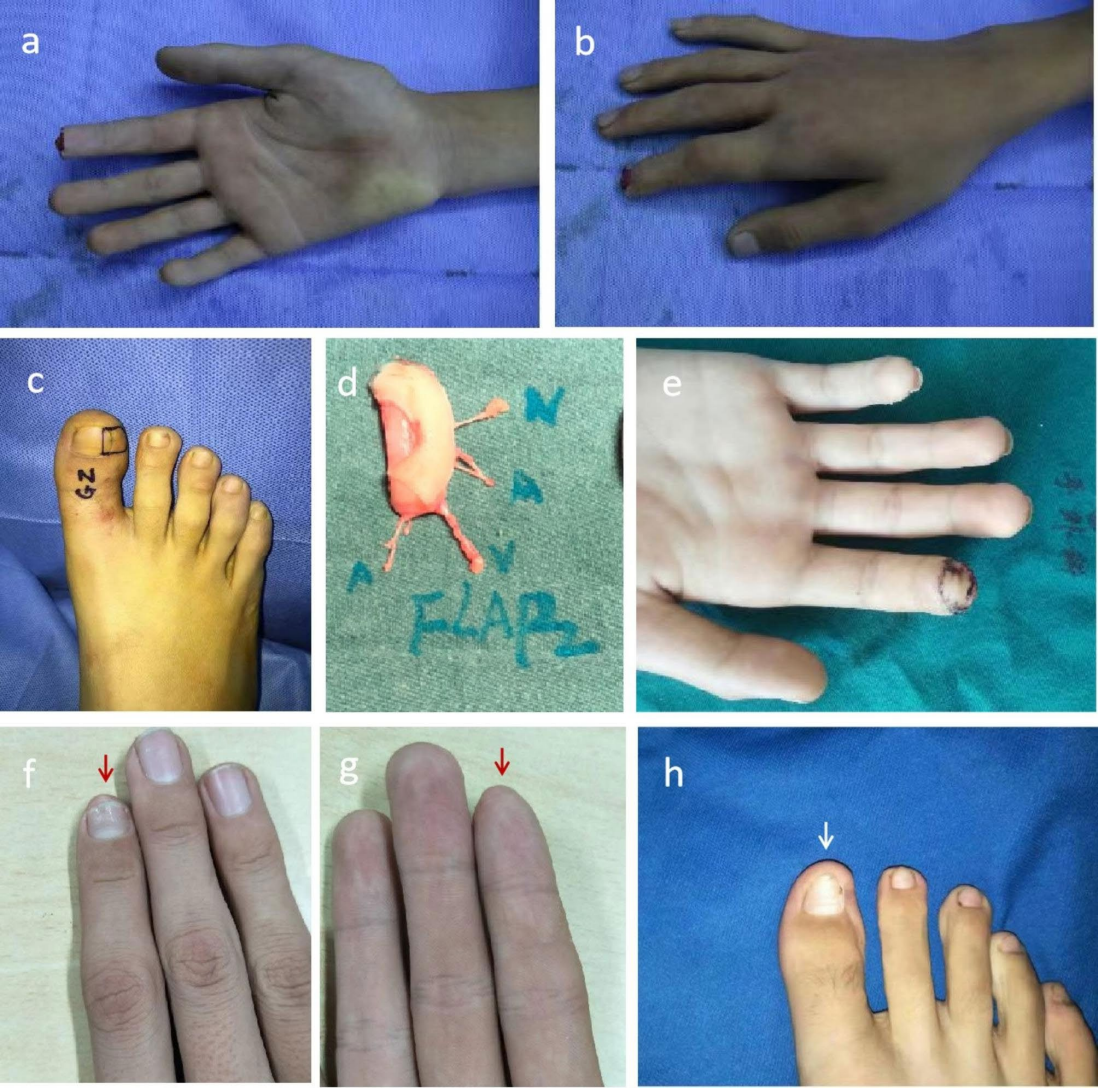
Mini hallux toenail flap pedicled with the hallux transverse artery and toe pulp vein for the fingertip injury of the right index finger. **(a-b)** Fingertip injury of the right index finger (Allen’s type II). **(c)** Equivalent design of donor area. **(d)** Free mini hallux toenail flap pedicled with the hallux transverse artery and toe pulp vein for transplantation. **(e)** Mini hallux toenail flap pedicled with the hallux transverse artery and toe pulp vein was transplanted to cover the defects of skin and nail bed during the operation. **(f-g)** The appearance of the injured right index finger 24 months after the operation. **(h)** The appearance of the donor area of the right hallux 24 months after the operation

A thirty-six years old female worker with a right index fingertip damaged by a door crush was sent to the emergency room within 1 h after injury. Emergency debridement was performed, finding that soft tissue defect and nail defect of the right index fingertip was about 0.8 cm × 1.2 cm and 0.8 cm × 0.7 cm, respectively, with intact phalanx (Allen’s type II). Then the miniature hallux toenail flap pedicled with the hallux transverse artery and toe pulp vein based on the “equivalent design theory” was used to reconstruct the damaged fingertip. The graft survived 14 days after the operation, and the damaged fingertip and donor site were beautiful, with good function 24 months after the operation. (Fig. 4)

## Discussion

It is very common in clinics that fingertip injury leads to skin and nail bed complex defects. As an important treatment modality to fingertip injury, conservative treatments, especially wound cleaning and dressing, seem to be popular and rejuvenated mainly in Europe in recent years [10.–11] Because fingertip wounds are possible to heal by secondary intentions without producing a scar [12], many scholars think that it is possible to treat fingertip injury by debridement and fingertip dressing effectively, and many studies [13–12] reported that wound cleaning and fingertip dressing therapy had excellent effects on fingertip amputation. Especially Semi-occlusive dressing, as a classic conservative treatment, has been confirmed to have prime clinical effects on fingertip amputation with 100% wound healing rate, lower pain, less cold sensitivity, less trophic changes in fingers and nails, and better 2-point discrimination [14]. But in our opinion, the largest drawback of the conservative treatment is the unaesthetically pleasing appearance caused by shortened digits and missing nails. The current study shows that mini hallux toenail flap pedicled with the hallux transverse artery and toe pulp vein transplantation technique not only has excellent clinical effects but also can reconstruct damaged fingertips aesthetically.

Traditional operations, such as random abdominal flap, local pedicled flap, or advanced flap, are mainly used to repair the fingertip wound. However, the repaired fingertips are not only bloated but also without nails. With people’s aesthetic requirements for such injuries, how to reconstruct the integrity of the skin and nail bed of the damaged fingertip and grow a beautiful nail is a clinical problem faced by surgeons. Many scholars have made active exploration in this field. Lee et al. [8] used a thenar fascial flap and subsequent nail bed grafting to reconstruct nail bed defects. Although this method has achieved good results, it needs to wait for the palmar fascia flap to heal, and full-thickness fingernail transplantation is performed in the second stage leading to a long course of the disease. In addition, the donor area comes from the palm, and scar formation increases the impact on the palmar aesthetics of the hand. Xing et al. [15] designed a rectangular flap in the nail epithelium, combined with the finger pulp, to promote the skin flap in thirty clinical applications. By increasing the exposure of the nail root from 0.3 to 0.4 cm, although the appearance of the nail was restored, the normal length of the nail and finger was still missing because the defects of the nail bed and fingertip had not been restored. Wang et al. [16] used bilateral free lateral hallux nail flap combined with the bone to treat seven distal interphalangeal joint defect cases with an average follow-up of 93.4 months. The results showed that the injured fingers had a good appearance and sensory and motor function. However, this procedure is only applicable in the cases of the complete distal phalangeal defect, and it is not suitable for the simple destruction of the soft tissue of the fingertip, including the finger pulp and the nail bed. Takmi Yamamoto et al. [17] applied improved free hallux pulp combined with adipose tissue flap transplantation to reconstruct the defective fingertip. The graft was placed horizontally in the receiving area, the hallux pulp was used to repair the finger pulp defect, and the adipose tissue was applied to cover the missing nail bed. The appearance of the regrew nail in this operation was still not ideal.

The transplantation of the hallux toenail flap to reconstruct the shape of nails has achieved good clinical results [18–20]. The traditional toenail transplantation takes the dorsal toe vein as the reflux blood vessel. In order to ensure the smooth return of the toenail flap vein, the toenail flap has to carry parts of the dorsal toe skin, which not only causes significant damage to the supply area but also causes the appearance of swollen fingers after surgery. Due to the limited defect of the fingertip pulp and nail bed caused by fingertip damage, we designed a mini hallux toenail flap with blood supplied by the transverse hallux artery and refluxed by the toe pulp vein according to the degree of defect. We realized the “equivalent design theory” of “what is missing, what is needed, how much is missing, and how much is needed” by simply cutting out part of the hallux pulp and nail bed for free transplantation, discarding excess surrounding skin, covering the defect area of the fingertip, and plastic repair of the skin and nail bed with fingertip defect. At the same time, by placing the hallux toenail flap-skin nail bed complex horizontally to cover the fingertip wound, the skin at the junction of the nail bed of the transplanted hallux toenail flap and the lateral edge of the hallux can form subungual skin of the reconstructed nail, the skin of the transplanted hallux toenail flap can be sutured with the skin edge of the nail stump to form a new nail contour, the nail bed of the transplanted hallux toenail flap can be non-invasive sutured with the nail bed of the recipient site, forming a nail bed close to the original size. Thus, it can promote the smooth growth of normal nails and eventually restore a fingertip with a beautiful appearance and approximate normal function.

In the results of this study, we found that the plastic repair of fingertip injury with an equivalent designed mini hallux toenail flap was safe, and the intraoperative blood loss was only 20.66 ± 4.87 ml. Although vascular crisis occurred in three cases, the transplanted hallux toenail flap ultimately survived after surgical treatments. The average nail growth time of 56 patients was 9.68 ± 2.11 months, all of which had a beautiful appearance without nail deformity and recovered to normal fingertip and nail contour. Semmes-Weinstein tactile pressure was 0.32 ± 0.14 g, and the TDP was 7.33 ± 1.02 mm with an excellent-good rate as high as 100%.

There are some advantages and disadvantages of mini hallux toenail flap pedicled with the hallux transverse artery and toe pulp vein based on the equivalent design theory for fingertip injury. (i) Simple anesthesia method: The operation is performed under the anesthesia of the finger or toe root, with less bleeding. (ii) Compound minimally invasive concept: According to the defect area of the fingertip, the hallux toenail flap is designed equally without carrying excess soft tissue and indeed realizes the “equivalent design theory” of “what is missing, what is needed, how much is missing and how much is needed”. After surgery, the pulp of the fingertip and nail bed are reconstructed. Through the natural growth of the nail, not only the complete shape of the regenerated nail and the perfect normal contour of the finger are achieved, but also the finger function is restored. The psychological burden and self-esteem of the injured patients are ultimately solved. (iii) The anatomy is simple: The donor area of the foot is less damaged and can be directly sutured to protect the bearing function and shape of the foot to the maximum extent. (iv) Recovery of sense: Because the nail bed complex tissue of the lateral skin of the hallux is small, the intrinsic nerve of the toe is difficult to remain in the nail bed complex of the lateral skin of the hallux. The free tissue is small without the nerve graft, and the sensory receptors are formed by themselves. (v) The most significant deficiency of the operation is that it requires high micro-surgery techniques of the surgeon. To prevent damage to the blood vessels, the free hallux toenail flap should be carried out under the microscope, which needs high micro-surgery techniques and intimate knowledge of the foot vascular anatomy of the surgeon. (vi) This technique only applies to Allen’s type II fingertip injury, and for other fingertip injuries with serious phalangeal fractures, the mini hallux toenail flap pedicled with the hallux transverse artery and toe pulp vein based on the “equivalent design theory” is not compatible in our opinion.

In addition, there are some precautions of the technique. (i) Strictly grasp the indications for surgery: This technique is applicable for the soft tissue damage of the fingertip combined with part defects of the nail body, which is not suitable for injury with many nail root defects and distal phalanx bone defects (Allen’s type II fingertip injury). (ii) Select the phalanx ventral vein of the hallux in the donor area: This operation only cuts the skin of the toe pulp, without any dorsal skin, and only uses the phalanx ventral vein as the reflux vein. Under the condition of using the lower limb tourniquet, the phalanx ventral veins are well-filled to facilitate searching. Because the donor area should be placed horizontally on the finger, the phalanx ventral vein should preferably be the lateral vein of the hallux, which is convenient for anastomosis with the digital ventral vein of the affected area. (iii) Because the skin and nail bed at the junction of the lateral edge of the hallux are very tight, the nail bed should be separated under a microscope to prevent damage to blood vessels. (iv) Non-invasive suture of the nail bed: Non-invasive suture line is used to perform an end-to-end smooth suture between the hallux nail bed in the recipient site and the fingernail bed stump in the donor area to minimize postoperative nail deformities. (v) Due to the slow growth of nails after transplantation of the mini hallux toenail flap, the nail bed must be kept moist after surgery, and erythromycin ointment can not only keep the nail bed moist but also play an anti-inflammatory effect.

In addition to being superior to previous toenail flap transplantation used in the clinic, mini hallux toenail flap pedicled with the hallux transverse artery and toe pulp vein transplantation technique may also be better than conservative treatments, including stump revision and skin flap repair ( as V-Y shape flap ) in our opinion.

## Conclusion

In conclusion, the plastic repair of fingertip injury with an equivalent designed mini hallux toenail flap pedicled with the hallux transverse artery and toe pulp vein has the advantages of a beautiful appearance and good finger function after surgery. Especially for Allen’s type II fingertip injury, the technique can restore a normal nail contour and fingertip with excellent clinical efficacy. No postoperative complications occurred in all donor areas, which is worthy of promotion and application in clinics.

## Data Availability

The datasets used and/or analysed during the current study are available from the corresponding author on reasonable request.
